# Voltage-gated ion channels as novel regulators of epithelial ion transport in the osmoregulatory organs of insects

**DOI:** 10.3389/finsc.2024.1385895

**Published:** 2024-05-21

**Authors:** Jocelyne Dates, Dennis Kolosov

**Affiliations:** Department of Biological Sciences, California State University San Marcos, San Marcos, CA, United States

**Keywords:** Malpighian tubules, salt and water balance, voltage-gated ion channels, ion transport, excretion

## Abstract

Voltage-gated ion channels (VGICs) respond to changes in membrane potential (V_m_) and typically exhibit fast kinetic properties. They play an important role in signal detection and propagation in excitable tissues. In contrast, the role of VGICs in non-excitable tissues like epithelia is less studied and less clear. Studies in epithelia of vertebrates and invertebrates demonstrate wide expression of VGICs in epithelia of animals. Recently, VGICs have emerged as regulators of ion transport in the Malpighian tubules (MTs) and other osmoregulatory organs of insects. This mini-review aims to concisely summarize which VGICs have been implicated in the regulation of ion transport in the osmoregulatory epithelia of insects to date, and highlight select groups for further study. We have also speculated on the roles VGICs may potentially play in regulating processes connected directly to ion transport in insects (e.g., acid-base balance, desiccation, thermal tolerance). This review is not meant to be exhaustive but should rather serve as a thought-provoking collection of select existing highlights on VGICs, and to emphasize how understudied this mechanism of ion transport regulation is in insect epithelia.

## 1 Introduction

Insects are the most diverse group of animals with approximately 1 million species that span all geographical, aquatic, and terrestrial environments. In order to overcome their physiological disadvantage of having a large surface-to-volume ratio, insects have evolved and conserved a diverse range of systemic osmoregulatory functions to maintain internal homeostatic conditions (1, 2) that protect against unfavorable environmental challenges (3). Many insects face frequent and rapid salt-and-water imbalance due to their environmental ion availability changes, feeding habits, alterations in acid-base balance, desiccation, changes in environmental temperatures, and even buoyancy.

For instance, a blood-feeding mosquito must get rid of extra water ingested with a blood meal rapidly to address the post-prandial salt-and-water imbalance. A caterpillar eating ~5 times its own body weight in food daily must excrete gargantuan amounts of metabolic wastes and plant-based xenobiotics and be able to adjust ion transport within minutes when necessary. To add insult to injury, the only way for a caterpillar to digest plant-based food and tap into the nutritional potential of plants is to raise midgut pH to ~11 to dissociate plant proteins from tannins (4). Some insects (e.g., beetles) possess a sophisticated “desiccation tolerant” physiological adaptation to cope with intermittent or evaporative water loss by accumulating and storing water in the haemolymph (2, 5, 6). Many insects face rapid and drastic changes in the temperature of their environment. Chill-susceptible insects experience chill injury or chill coma with exposure to unfavorable thermal conditions when key osmoregulatory and active ion transport mechanisms decline, disturb membrane polarization and ion balance, and negatively impact energy budgets as temperatures decrease (1, 7, 8). *Chaoborus* midge larvae control buoyancy by manipulating pH levels using ion-transporting endothelia adjacent to the air-sac’s pH-sensitive protein resilin, which encourages the passive diffusion of gases across the endothelium that covers air-sac (9). All of these processes involve epithelia/endothelia that may benefit from employing fast ion transport-regulating mechanisms like the one discussed in this review.

Epithelial tissues simultaneously serve as: (i) a barrier between internal and external environment, and (ii) as a conduit for selective exchange of ions, nutrients, and wastes. Integral membrane proteins, like ion pumps, channels, aquaporins, and transporter are embedded in the membrane and form complexes that regulate V_m_ (10, 11) and provide a transmembrane pathway for ions and fluid (12).

Ion channels can be activated by temperature, osmolarity, ligand binding, mechanical change in the cell membrane, pH, neuroendocrine signals, and V_m_ (13–15) and display a spectrum of ion selectivity (Na^+^, K^+^, Cl, and Ca^2+^) (16). For the purpose of this review, voltage-gated ion channels (VGICs) are broadly defined as channels belonging to the voltage-gated ion channel superfamily regardless of whether they’re actually activated by changes in V_m_, which is considered on a case-by-case basis. The primary function of VGICs is to generate action potentials in excitable tissues in response to changes in the cell V_m_ (17–23). Since the development of patch-clamp electrophysiology, protein purification and molecular and biochemical techniques, >250 types of ion channels have been identified (24, 25).

Interestingly, expression of many Ca_V_, K_V_, Na_V_, and non-selective cation permeable VGICs has been reported *specifically in epithelia* of animals ranging from early divergent Placozoans to vertebrates (e.g., 26) (See **Table 1**). The genesis of the study of how VGICs regulate ion transport in insect epithelia traces itself to a recent collection of studies that investigate the topic in the Malpighian tubules (MTs) of lepidopterans. Analogous to the mammalian kidney, the MTs of insects, together with the hindgut, serve as the primary osmoregulatory and excretory organs carefully balancing the uptake and recycling of vital ions and fluid while efficiently excreting ingested xenobiotics/toxins, and metabolic wastes (50).

**Table 1:** Voltage-gated ion channels (VGICs) expressed in non-insect epithelia with potential roles they may play noted (where available).

Animal clade	Species	Tissue/Organ	Differential Factor/Proposed Role	VGICs	Ref.
**Placozoa**	*Trichoplax adhaerens*	dorsal epithelium	–	**Ca_V_1**	(27)
		outer edge of dorsal epithelium	–	**Ca_V_2**	(28)
**Asteroidea**	*Patiria pectinifera*	coelomic epithelium	–	**Ca_V_3, HVCN1, TRPA, TRPM**	(29)
**Mollusca, bivalves**	*Tridacna squamosa*	gill/ctenidium	light exposure	**Ca_V_1**	(30)
	*Crassostrea gigas*	mantle epithelium	salinity, exposure to dilute seawater	**Ca_V_3**	(31)
**Teleosts, eels**	*Anguilla japonica*	gill epithelia	environmental salinity (freshwater vs seawater)	**PVC - SCN3B, Ca_V_1, TRPA1, H_V_CN1; MRC -Ca_V_a2d3**	(32)
	*Anguilla anguilla*	swim bladder epithelium	metabolic activity (rest vs. exercise)	**Ca_V_1, Ca_V_2, Ca_V_3, Ca_V_a_2_d_2_, Ca_V_β, CLCN, HCN, HVCN1, KCNA, KCNB, KCNC, KCND, KCNF, KCNG, KCNH, KCNMA1, KCNN, KCNQ, KCNT, KCNV, Na_V_, TRPC, TRPM**	(33)
**Amphibia**	*Rana esculenta*	Basolateral in distal convoluted tubule and intercalated cells of collecting duct in kidney nephron	K^+^ secretion/reabsorption	**KCNQ1**	(34)
**Chondrichthyes**	*Squalus acanthias*	Rectal gland	function unclear	**KCNQ1**	(35)
**Mammals**	*Homo sapiens*	HK-2 kidney epithelial cells	cytokine TGF-β1 stimulation	**HCN, KCNA, KCNH, KCNMA1, KCNQ, KCNS, TRPA, TRPC, TRPM, TRPV**	(36)
		Intestinal and gastric, skin, lung, liver, and kidney epithelia	–	**Ca_V_1**	(37)
		Vascular endothelium	K_V_ channels contribute to K^+^ transport	**Irk/Kir, BK, TRPC**	(38)
		lung epithelia	H^+^ secretion	**H_V_1**	(39)
		adrenal gland, lung	function unknown	**K_V_4.3/erg**	(40)
	*Mus musculus*	distal lung epithelium	development	**Ca_V_β, CLCN, KCNA, KCNH, TRPM**	(41)
		collecting duct epithelial cells	–	**Ca_V_β, CLCN, KCNQ, KCNS, TRPM, TRPV**	(42)
		Gastric, thyroid, intestinal and choroid plexus	K^+^ transport	**KCNQ channels**	(43)
		kidney nephron	K^+^ reabsorption and recycling	**KCNQ1**	(44)
		kidney, stomach, exocrine pancreas	K^+^ secretion and recycling, in maintaining the resting potential, and in regulating Cl^-^ secretion and/or Na^+^ absorption	**KCNQ1**	(45)
		kidney and colon epithelium	K^+^ secretion	**BK**	(12)
		primary cilia in renal epithelia	osmotic stress response	**TRPM3**	(46)
	*Canis lupus familiaris*	cultured kidney epithelia	osmolality stress	**SCN1B; Ca_V_2.3, 3.1; KCNQ4, KCNC4, HCN2, TRPV1,2; TRPM6**	(47)
			salt stress	**SCN1B; Ca_V_3.1; KCNQ4; KCNC3; HCN2; TRPC1; TRPV1,2**	
	*Didelphis virginiana*	opossum kidney (OK) cells	maturation	**SCN9A; CACNA1C; CATSPER2; CATSPER3; KCNAB2; KCNB2; KCNQ2; KCNH4; HVCN1; TRPM7; TRPM8; TRPC3; PKD2,1L2; TRPV4; HCN2**	(48)
			orbital shear stress	**SCN9A; CACNA1C; CATSPER2; CATSPER3; KCNAB2; KCNB2; KCNQ2; KCNH4; HVCN1; TRPM7; TRPM8; TRPC3; PKD2, PKD1L2; TRPV4; HCN2**	
	*Oryctolagus cuniculus* and *Ratus norvegicus*	kidney and colon	ion transport and stabilization of the resting membrane potential	**K_V_1.3/Shaker**	(10)
	*Ratus norvegicus*	pancreas, intestine, airway, kidney	provides the driving force for Cl^–^ transport across basolateral membrane of pancreas, airway, and intestinal epithelia	**KCNQ1**	(49)

VGICs in red decrease in mRNA abundance, while VGICs in purple increase in mRNA abundance (where available).

**Figure 1:**
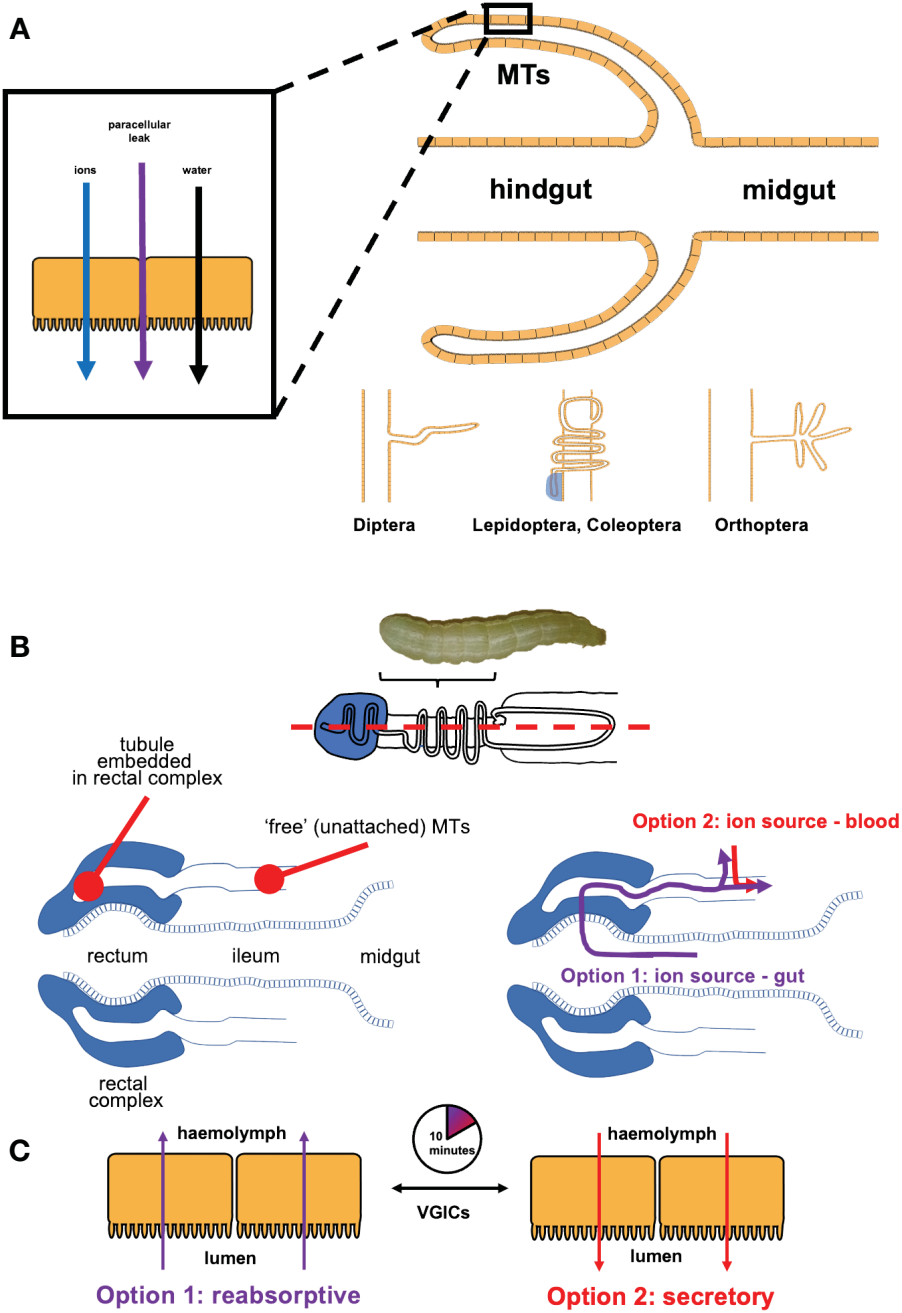
**(A)** Malpighian tubules (MTs) are blind outpouchings between the midgut and the hindgut of an insect - they secrete fluid by secreting ions into the lumen, and allowing water to follow by osmosis, with a paracellular junction leak component. Structure and functional adaptation depends on the insect clade. **(B)** In the larval lepidopterans, the distal end of each tubule is embedded into the rectal complex (in longitudinal section, dashed blue line, top panel). Each embedded tubule connects to the ‘free’ (unattached) region, suspended in the hemolymph and closely juxtaposed to the gut. Larvae have two options as a source of ions and water for secreting fluid in their MTs. The reabsorptive option (in purple) sources ions and water from the gut by way of the blind embedded tubules when the caterpillars are well fed. Some ions reabsorbed from the gut are transferred into the hemolymph across the unattached MTs. The secretory option (in red) sources ions and water from the larva’s hemolymph, when dietary ions and/or water are unavailable or are in short supply (e.g., postprandially or during molting). **(C)** The switchover between reabsorption and secretion takes as little as 10 min.

One phenomenon is common to all insect MTs – they must secrete ions into their lumen to osmotically draw water in, and drive excretion and osmoregulation (see **Figure 1**) (51). Evolving approximately 350 million years ago, the highly conserved two-cell-type structure of insect MT epithelium, comprised of stellate cells (SC) and principal cells (PCs), employ physiologically distinctive cell-specific transepithelial cation and anion transporting mechanisms (2). Although, the MTs of *Drosophila melanogaster* have been used as the insect model of study for human renal pathologies (52, 53), there are substantial differences in complexity of the insect MTs between different clades of insects (54), highlighting the need for clade-specific investigation of ion transport mechanisms in osmoregulatory epithelia (see **Figure 1A**). For instance, how PCs and SCs transport ions does not translate between insect clades, and in many cases blind ends of MTs are either closely associated with the hindgut or are embedded into a specialized structure (15, 55, 56).

In lepidopteran larvae, the distal end of MT is embedded into a specialized structure, the rectal complex (see **Figure 1B**). This allows the larvae to extract ions and water from their diet that they use to secrete fluid in the MTs. The remainder of the tubule (not embedded into the rectal complex) reabsorbs ions. However, when the gut is empty (e.g., during moulting or cessation of feeding), ions and water cannot be procured from the gut. A downstream region called the distal ileac plexus rapidly switches from ion reabsorption to ion secretion to enable osmotic secretion of fluid into the MTs, and to ensure that MTs function is not interrupted. This process is regulated in part by VGICs, which likely contribute to how rapidly (~10 min) the switch takes place in the MTs (**Figure 1C**). Given profound differences in the structure and function of MTs in insect clades, this begged the question of whether VGICs may be present in the MTs of other insects? Perhaps, even in other osmoregulatory epithelia of insects?

In addition to the MTs and the hindgut, many insects employ epithelia/endothelia that either play a direct role in osmoregulation (e.g., anal papillae of mosquitoes (e.g., 57, 58), collophore epithelia of springtails (e.g., 59)), or employ ion transport that is tied to other functions - e.g., gas bladder endothelia that regulate buoyancy in midge larvae (e.g., 9), blood-brain barrier endothelia that maintain the ion content of neuron-bathing fluid (e.g., 60). Caution must be used, however in the study of whether VGICs regulate ion transport in many of these organs, as excitable tissues layers (nerves, muscles) may confound results obtained in whole-organ studies.

Lastly, MTs epithelia play important roles in the immune response, oxidative stress, and response to xenobiotics and toxins (61). A speedy response to any of these using fast-acting VGICs would surely be of benefit to insects.

## 2 Voltage-gated Ca^2+^ channels

Ca_V_ channels are selectively permeable to Ca^2+^ and open in response to membrane depolarization (62, 63). In the osmoregulatory epithelia of insects, Ca_V_1 has been detected in the MTs of *Drosophila melanogaster*, and implicated in intracellular signaling, directional Ca^2+^ transport and the regulation of diuresis (64). Likewise, Ca_V_1 was shown regulate epithelial ion transport in the MTs of larval *Trichoplusia ni* (65), and larval and adult *Aedes aegypti* (58). Additionally, the same study has localized Ca_V_1 in hindgut epithelium of the blood-fed adults of *Aedes aegypti*, where it may play a role in the regulation of post-prandial diuresis. The function of other Ca_V_ channels in insect epithelia remains unclear. In most animals studied to date, Ca_V_ channels demonstrate conserved biophysical properties across clades, and Ca_V_2 has different properties from Ca_V_1 and Ca_V_3 (66). The use of isoform-specific inhibitors may help discern the roles of other Ca_V_3 isoforms in the MT of insects. Typical V_m_ of epithelial cells in MTs varies depending on the insect clade and osmoregulatory status of the animal (e.g., post-prandial, ion-rich diet). The use of high- and low-voltage activated Ca_V_ channels may provide an additional link between the regulation of ion transport and Ca^2+^ signaling. Ca^2+^ as a second messenger whose role is to maintain Ca^2+^ stores and concentration, a powerful activator of potassium transport mechanisms (14).

In addition, as Ca^2+^ is a general second messenger, there is potential for Ca_V_ channels to be involved in the regulation of epithelial processes other than ion transport, such as acid-base balance, desiccation and thermal tolerance, buoyancy control, and regulation of blood-brain barrier.

## 3 Voltage-gated K^+^ channels

K_V_ channels belong to a diverse superfamily of proteins (67, 68) distinguished by their K^+^ selectivity (21), and their role in K^+^ transport, recycling, and intracellular signaling (69–71). Within the K^+^ channel superfamily, K_V_ channels and at least 9 of their subfamilies are widely expressed in the membranes of excitable and non-excitable tissues (10, 72). K_V_ channels can also be activated by Na^+^ or Ca^2+^, can be inwardly rectifying or delayed rectifying, and display fast or slow kinetics of opening and closing (68, 73–76). K_V_ channels (K_V_1-K_V_4) named Shaker/K_V_1.3, Shab/K_V_2, Shaw/Kv3.1, and Shal/K_V_4, based on *Drosophila* studies, vary in biophysical properties (77–82).

Inward-rectifying potassium (Kir) channels received their name from the fact that the inward flow of K^+^⁠ into the cell at any given voltage is greater than the outflow at same voltage but opposite in polarity (83). Kir channels play a role in K^+^⁠ secretion into MTs of the yellow fever mosquito *Aedes aegypti*, the fruit fly *Drosophila melanogaster*, and larval lepidopteran *Trichoplusia ni* (84). In *Drosophila*, three Kir homologues irk1, irk2 and irk3 are involved in PC-based K^+^⁠ secretion (85, 86). In the MTs of yellow fever mosquito *Aedes aegypti*, transepithelial secretion of K^+^ is regulated by Kir1 located in the SC’s basolateral membrane and by Kir2B in the basolateral membrane of PC’s (87, 88). Together Kir1 and Kir2B are credited with conducting 66% of total transepithelial K^+⁠^ secretion in *Aedes* MTs. Additionally, Kir1 has been shown to play a role in K^+^ secretion in the basolateral membrane of principal cells of a larval lepidopteran *T. ni* (55). Notably, there are pronounced differences in cellular localization of Kir isoforms in the MTs of different insects but have been detected in the MTs of larval lepidopterans using transcriptomic approaches (56, 89).

KCNQ/K_V_7.1 are VGICs found in excitable and non-excitable cells that are sensitive to extracellular [K^+^], providing constant repolarizing force that controls V_m_ (21). Interestingly, the membrane protein Potassium Voltage-gated Channel Subfamily E Regulatory Unit 3 (KCNE3) regulates the function of K^+^ channel KCNQ1 channel by co-assembling with its ɑ subunits preventing channel closure and converting it into a voltage-independent channel (90), while remaining constitutively active and open (43) at a negative voltage that would typically close the channel (91, 92). In insect epithelia, KCNQ1 was found to be enriched specifically in Drosophila MTs, although its function in the renal tissue has not been investigated to date (93). Recently, several studies detected KCNQ channels in MTs of larval lepidopteran *T. ni* (89, 94). KCNQ1 has also been detected in the MTs of larval dipteran *Ae. aegypti* where this channel may contribute to K^+^ secretion in the MTs of brackish water (BW) larvae, helping the larvae rid themselves of extra K^+^ and preventing K^+^ loading with BW exposure. KCNQ1 was also found in anal papillae of larval *Ae. aegypti*, where it may play a role in uptake of environmental K^+^ by the AP of larvae in freshwater (FW), aiding larvae in retention of hemolymph K^+^ in the face of diffusional K^+^ loss to FW (58).

In addition to being directly involved in ion transport in osmoregulatory epithelia, K_V_ channels may be involved in response to extracellular hyperkalemia observed in chill-injured insects (e.g., 95). Low levels of K^+^ are essential for the proper function of the brain in insects (96), and potential use of K_V_ channels by the blood-brain barrier endothelia (especially in K^+^-feeding herbivores) may be advantageous for rapid rebalancing of K^+^ between body compartments.

## 4 Voltage-gated Na^+^ channels

The ability to rapidly regulate natriuresis may be advantageous to certain insects – e.g., the female mosquito can rapidly increase diuresis to efficiently remove excess Na^+^ post ingestion of a blood meal (97). In blood-feeders, the electrochemical potential of Na^+^ is what drives Na^+^-rich fluid secretion (98). Transcripts encoding Na_V_ channels like Para and Nalcn have been detected in the MTs of larval lepidopterans *T. ni* (89, 94) and *Helicoverpa armigera* (99) as well as that both MTs and anal papillae of larval dipteran *Ae. aegypti* (58). Previous pharmacological studies suggested that apical Na^+^ channels regulated Na^+^ uptake in anal papillae (57, 100) and Na^+^ channels present in the MTs of *Ae. aegypti* transport Na^+^ from haemolymph to lumen via voltage gradients created by V-type H^+^ ATPase (88, 101, 102). A recent study demonstrated that Nalcn is present in the water-facing membrane of anal papillae in the larval *Ae. aegypti* (58). Although a significant number of neuropeptide toxins specific to Na_V_ channels have been identified among vertebrates, insect Na_V_ channels are remarkably different, which may prove mechanistic study of their function in insect epithelia rather challenging (103).

Na^+^ balance is especially important to plant-feeding insects as all excitable tissues require Na^+^ for producing action potentials, but it can be quite low in the diet, necessitating bizarre behaviors like puddling in adult moths aimed at supplementation of low dietary Na^+^ with that acquired from mud puddles of vertebrate urine. Thus, in addition potentially contributing to epithelial Na^+^ transport Na_V_ channels could be used by blood-brain endothelia to quickly rebalance Na^+^ between body compartments.

## 5 Cation-selective VGICs

### 5.1 Transient receptor potential channels

TRP channels are cation-permeable (Na^+^, K^+^, Ca^2+^) voltage-dependent channels (104). TRP channels are activated through a range of gating mechanisms including V_m_ depolarization (105). TRPA channels carry out functions in many excitable and non-excitable tissues like those found in *Drosophila* gut epithelia (106, 107). Several families of TRP channels have been reported in the osmoregulatory epithelia of in insects, including insect-specific Painless and Pyrexia TRPA channels. TRPA channels have been detected in MTs of larval *T. ni* (89), *Pieris rapae* (108) and *Bactrocera dorsalis* (109). TRPA1, TRP/Painless and TRP/Pyrexia are more permeable to K^+^ than to Na^+^, with well-established roles in nociception and thermotaxis in excitable tissues of insects (110–112). Six TRPA channels have been detected in MTs and AP of larval *Ae. aegypti* and mRNA abundance of every channel was altered as a result of BW exposure in MTs or anal papillae (58). TRP channels may provide an additional link between epithelial V_m_, ion transport, Ca^2+^ signaling, and activation of other VGICs in insect MTs.

In addition to playing a role in the regulation of epithelial ion transport, TRP channels like Painless may serve as peripheral thermal sensors since they have been shown to respond to increased temperatures *in vitro* (i.e., regardless of the context of which tissue they’re expressed in) (113). Attuning the function of osmoregulatory epithelia to changes in environmental temperature may be particularly advantageous for these small ectothermic animals.

### 5.2 Hyperpolarization-activated cyclic nucleotide-gated channels

Whereas most VGICs open in response to depolarization, a stand-out group of VGICs is activated by hyperpolarization - the HCN channels, which are permeable to both Na^+^ and K^+^, and are additionally activated by cyclic nucleotides, where the latter overrides dependence on V_m_ changes (114). In osmoregulatory epithelia of insects, the HCN channels have been detected in the MTs of larval lepidopteran *T. ni* (57, 58, 88, 89, 94) and when HCN1 channels are blocked in MTs, ion transport switches direction from K^+^ secretion to K^+^ reabsorption (89). Cyclic nucleotides are known to alter fluid secretion in the MTs of larval and adult insects (e.g., 115–117). HCN channels can provide an additional link between direct activation of ion transport and second messenger-based hormone action.

## 6 Select unstudied VGICs detected in osmoregulatory epithelia of multiple insects

Transcriptomic studies on osmoregulatory epithelia of lepidopterans and dipterans uncovered the presence of many more VGICs (e.g., H_V_, Cl_V_, BK, KCNA, KCNC, KCNS, TRP M, TRP V channels), expression of which has not been confirmed using direct molecular, protein-based, or pharmacological approaches to date (26, 58, 65, 89, 94). Whether these VGICs play a role in the regulation of ion transport in insect epithelia remains unexplored. H_V_ and Cl_V_ channels may play a role in acid-base balance regulation since acid H^+^ transport can contribute to acid equivalents, and Cl^-^ is often transported by epithelia in exchange for HCO_3_^-^ – to the best of our knowledge, these have not been examined in osmoregulatory epithelia of insects to date. TRP channels may serve as peripheral sensors of allelochemicals and xenobiotics in herbivores as many TRPs are activated by noxious plant-based chemicals, as well as peripheral temperature sensors (see above). Many TRP channels are volume-sensing and mechanosensitive (118), both of which would offer insect epithelia an additional mechanism for fine-tuning their ion transport rapidly.

## 7 Discussion

### 7.1 Current gaps in research – what remains to be explored

#### 7.1.1 Are VGICs connected with mechanosensation in epithelia?

The role of VGICs in excitable tissues of animals has been well established (37). In insect epithelia however, VGICs seem to play an integral role in the regulation of ion transport and of V_m_ yet studies on this topic remain largely scarce.

A likely advantage of the presence of VGICs in animal epithelia is the ability to quickly respond to changing ion concentrations. In the MTs of insects, this may be relayed via mechanosensation of fluid flow and hydrostatic pressure, which may result from alternating bouts of diuresis and anti-diuresis, changing epithelial cell volume by applying pressure to the cells against the basal lamina that encases the MT epithelia. Mechanosensitive ion channels found in insect epithelia (e.g., 58, 89, 94) can detect mechanical changes in the epithelial cell membrane caused by changes in fluid flow, hydrostatic pressure and cell volume, and open resulting in changes in V_m_ and setting off intracellular second messengers (cAMP, Ca^2+^) cascades (119). VGICs have been shown to respond transcriptionally to mechanical stress at least in some epithelia and can be used to amplify this signal (see **Table 1**). An instance of this has been described in intestinal epithelia, where TRPM5 channel triggers membrane depolarization in response to nutrient levels and opening of Ca_V_ channels amplifies the Ca^2+^ signal.

#### 7.1.2 How are VGICs activated in insect epithelia?

Mechanisms of VGIC activation in insect epithelia remain largely unexplored. Peptide toxins extracted from metazoan venoms that target the specific subtypes of animal VGICs may prove to be a useful pharmacological tool to gaining a better understanding of insect VGICs (103). Do VGICs respond directly to the changes in epithelial cell V_m_ resulting from altered intracellular and extracellular ion concentrations? Are VGICs activated by other upstream mechanisms (e.g., mechanosensitive and/or ligand-gated ion channels)? When luminal fluid flow decreases, cell volume may increase, activating mechanosensation and recruiting additional directional ion transport. MTs of insects have recently been shown to react to changes in hydrostatic pressure (120). Ligand-gated ion channels have similarly been detected in the MTs of multiple insect clades (e.g., 121, 122) and epithelial V_m_ can change in response to stimulation of neurotransmitters and endocrine ligands. VGIC can also be used to amplify activation of ligand-gated ion channels.

#### 7.1.3 Do VGICs remain voltage-gated in epithelia?

Voltage sensitivity varies between the different types of VGICs with K_V_, Na_V_, and Ca_V_ displaying high sensitivity, activating and opening following membrane depolarization (43), and TRP channels demonstrate comparatively low voltage sensitivity (16). It is widely accepted that the biophysical properties of VGICs, although conserved in the same channel across many taxa, may be modified by channel subunit assembly for VGICs that are made up of multiple subunits. For instance, K_V_ ɑ subunit complexes can also co-assemble with β subunits of K_V_ channels creating isoforms with different biophysical properties (19, 21, 22, 72, 90, 123).

## 8 Conclusions

It has been decades since the first VGIC was reported in animal epithelia. Recent studies in osmoregulatory epithelia of insects point to an abundance of VGICs, several of which have already been demonstrated to regulate ion transport. Further study of the roles specific VGICs play in the regulation of ion transport in insect epithelia may prove a fruitful ground for basic knowledge needed for instance to design better targeted integrated pest management strategies.

Important questions remain about the role of VGICs in the osmoregulatory epithelia of insects. Do VGICs demonstrate the same ion selectivity in epithelia as they do in excitable tissues? Do VGICs directly contribute to directional ion transport? Do VGICs establish the driving force for ion transport? Do VGICs establish/set V_m_ in insect epithelia? Do VGICs assemblages in insect epithelia differ based on external salinity, dietary ion availability, and ions used to drive diuresis in MTs (e.g., K^+^-eating herbivores vs Na^+^-eating omnivores/carnivores/puddlers)? Are VGICs used to regulate processes connected to the osmoregulatory function in insects – e.g., acid-base balance, desiccation, thermal tolerance, buoyancy control, regulation of blood-brain barrier?

## Author contributions

JD: Conceptualization, Writing – original draft, Writing – review & editing. DK: Conceptualization, Data curation, Formal analysis, Funding acquisition, Investigation, Methodology, Supervision, Visualization, Writing – original draft, Writing – review & editing.
